# Beyond biomimicry – next generation applications of bioinspired adhesives from microfluidics to composites

**DOI:** 10.3762/bjnano.15.79

**Published:** 2024-08-05

**Authors:** Dan Sameoto

**Affiliations:** 1 Department of Mechanical Engineering, University of Alberta, Edmonton AB, T6G 1H9, Canada

**Keywords:** adhesion, biomimicry, composites, gecko, robotics, soft lithography

## Abstract

In this perspective article, Professor Dan Sameoto outlines his opinion on future opportunities in the field of biomimetic adhesives. Despite over twenty years of excellent academic work by groups all around the world in this subfield, the economic value and impact of these materials is somewhat underwhelming. The question for the field is whether it should have a scientific and engineering focus to create every greater performance and understanding of the materials and hope that “if we build it, they will come”. Perhaps we should expand our concept on what could be the desirable end applications for such materials and focus efforts on finding better end applications in which these materials can truly shine; a few of those applications like microfluidics and composites are highlighted in this article. It is time for a next generation of research to look beyond biomimicry and look towards re-engineering applications to make use of these materials’ unique properties in economically viable ways.

## Perspective

As of the time of this writing, it has been 24 years since the seminal work by Kellar Autumn and his colleagues demonstrated how a single gecko foot hair could generate adhesion [1]. Autumn’s discovery that van der Waals forces were the primary mechanism behind the extraordinary climbing capabilities of geckos launched over 20 years of intense scientific and engineering efforts to understand and industrially mimic this technology [2–4]. Coincidentally, in 2000, the same year that Autumn’s paper was published, the Nobel Prize for physics was awarded to Zhores Alferov and Herbert Kroemer, and Kroemer’s Nobel lecture that year included the statement that “the principal applications of any sufficiently new and innovative technology always have been – and will continue to be – applications created by that technology” [5]. I often come back to that statement by Dr. Kroemer and try to ask myself: Has what we have achieved with biomimetic adhesives met that criteria? The honest answer at the moment is no, but perhaps it is still possible; even Velcro® took approximately 20 years from invention to wide commercial acceptance. The difficulty with biomimetic adhesives is that they are competing in a crowded market of existing technological solutions. Existing pressure-sensitive adhesives [6] are available in a wide range of tackiness, use cases, and temperatures; also, they are relatively inexpensive as they are usually designed for one-off uses. Hook and loop fasteners [7] and magnets are much better in terms of reliability and adhesion force than current biomimetic materials but need mating surfaces that are compatible. Traditional fasteners like screws, bolts, and nuts are available for assemblies that do not need to be disconnected frequently but are extremely strong compared to all other reversible bonds. So, when biomimetic adhesives are applied as tape, reversible adhesives, or fasteners, they are competing in a crowded marketplace in which they do not have clearly superior performance to overcome incumbent advantage. Therefore, we must think outside the box in terms of new applications for biomimetic adhesives for truly substantial industrial impacts to be made. These new application areas are my primary research focus today; it makes the efforts of all those who have participated in the academic and industrial development of the field more valuable. How can these biomimetic materials possibly serve a niche not currently fit by any existing technology or enable applications otherwise not possible with existing adhesives?

In this perspective article, I will cover three unique subtopics that our group has advanced that can be substantially improved through the use of biomimetic adhesives. These subtopics are microfluidics, soft robotics, and reconfigurable composites. Microfluidics involves the manipulation and flow of fluids on a very small scale, typically with nanoliters or less of fluid and with feature sizes ranging from a few hundred microns down to sub-micrometer size [8–9]. It has been an active area of academic research for well over 30 years. Microfluidics technology has eventually enabled a variety of new innovations, including COVID-19 rapid tests [10], microfluidic displays [11], and low-cost diagnostics [12]. Soft robotics is a newer academic topic that has gained much popularity since approximately 2010 [13] and involves using very deformable materials like elastomers to build robotic sensors, actuators, and even simple logic circuits [14] that use nearly exclusively or primarily soft materials. Soft robotics can be, in theory, very compliant and, thus, compatible with collaborative robotics, wearable components, and relatively safe human–robot interactions, which potentially provides a unique capability compared to traditional rigid robotics. However, there is a trade-off in soft robotics between the compliance of the material and the degree of controllability or even its ability to self-support under gravitational loads; thus, there is a major interest in stiffness-tunable materials for soft robotic systems [15]. This stiffness tuning in many cases relies on the temporary bonding of composite layers or materials to change effective stiffness. While soft robotics have shown a need for stiffness-tunable materials, the ability for composites in and of themselves to be reversibly bonded potentially opens up a far greater industrial impact and applications in adaptable, smart materials. Even improved sustainability could be achieved if laminates and composites could be reformed and reused. The secret to improve use cases of reversible adhesives and to improve applications in the above three subfields is to use biomimetic materials to manufacture composites which, in turn, have the capacity to change their stiffness, shape, or other mechanical properties of interest to fit the needs of soft robotics, microfluidic systems, or others.

**Figure 1:**
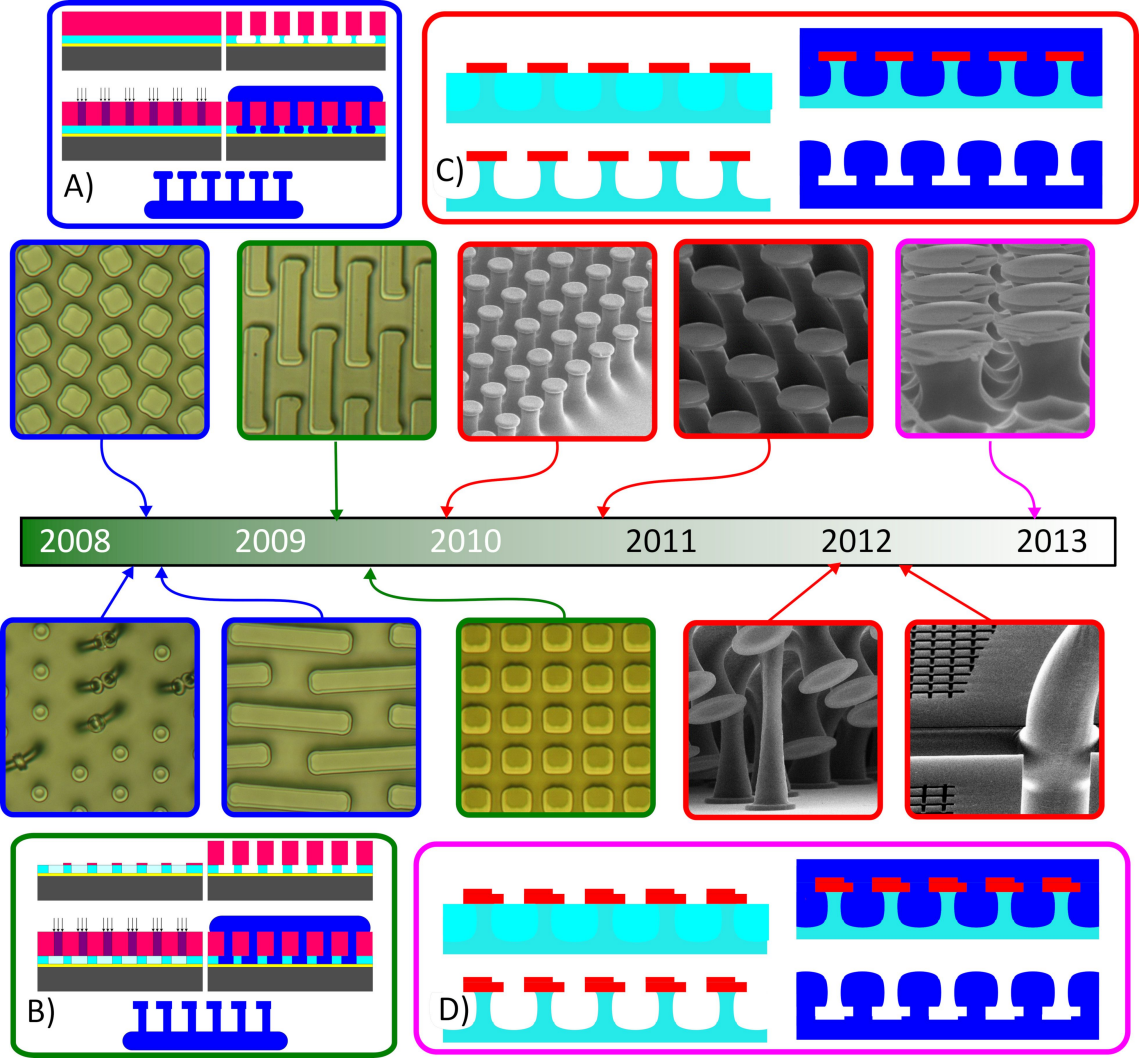
Chronological development of different gecko adhesive manufacturing processes that were developed by the author and then adapted by future students. Process (A) and (B) used silicone rubbers as the structural material for adhesives, and were capable of isotropic (A) or directional (B) adhesive performance depending on the symmetry of the cap. A more durable and useful system used deep-UV exposure of acrylic to form master molds, followed by a negative mold in silicone rubber (C, D). Isotropic designs were initially produced with polyurethane, and then structural materials were swapped to thermoplastic elastomers, with directional adhesion created by deliberate defects in the caps (D). Electrically conductive composite versions and geckofluidics were created in early 2012, and the focus was then switched to specific applications integrating biomimetic adhesives rather than adhesive development.

All of such applications necessitate that the biomimetic adhesives are robust, relatively inexpensive, and highly effective at adhering to different surfaces. Several corporations, including Setex [16], Gottlieb Binder [17], and a few others, have largely addressed these requirements for artificial biomimetic adhesives in the last decade, but the price per area is still far higher than that of competing adhesive solutions. At the time of writing, Setex has sold their industrial adhesive work to Shin-Etsu in Japan, and the Setex adhesive sheets are sold out on their website at a price of approximately $0.5/in^2^. A similar area of Velcro is closer to $0.1 (when including both halves), and strong industrial tapes like duct tape are closer to $0.02/in^2^, but can be as little as a fraction of a cent per square inch for packing tape sold in bulk. The price of materials from Gottlieb Binder is harder to find (requiring direct inquiries via their website), but, as they were constructed from silicone rubbers, the base materials costs can already be fairly high. If adhesives are made from Sylgard 184 (a very common structural material for biomimetic adhesives in academic literature and our previous publications), the list price is close to $400 CDN/kg at the time of writing (≈$300 USD). For an adhesive sample with an average thickness of 0.1 mm, this would represent approximately $0.19 of material alone and, therefore, the lowest possible cost. Alternative structural materials like styrene-ethylene-butylene-styrene (SEBS) can be purchased in bulk for as low as $5/kg and also can be microstructured in seconds via thermo-compressive molding, whereas Sylgard 184 requires at least a minute to cure even at highly elevated temperatures [18]. Both speed of molding/demolding cycles and raw materials costs are critical to the base cost of adhesive structures, so thermoplastics have both factors in their favor, even if the structural materials are inferior for specific use scenarios. I myself had an early focus on enhancing the manufacturability and adhesion performance of these adhesives [19] as have others since then [20] because commercial viability needs production rates and cost per part on par with hook and loop fasteners at a minimum. My work with biomimetic adhesives was begun in 2008 at Simon Fraser University with an intended end application in space robotics [21–22] (Figure 1), where the adhesives needed to adhere to surfaces under vacuum conditions with minimal preload and maintain strong adhesion in all directions. Initially, I focused on a variety of micro-tread structures with the expectation that improving the maximum aspect ratio before fiber collapse would be the best way to enhance adhesive performance, but very shortly after I made changes to the manufacturing process to achieve the mushroom-shaped cap reported by others to produce far higher adhesive strength [23–24]. These unusually designed biomimetic adhesives were discarded in favor of the more well-known and well-characterized mushroom-shaped fiber structures, and many groups developed similar shapes around the same time [2]. I will not cover the exact mechanisms by which these mushroom shapes have proven effective, as several review articles have already been published on this topic [2,4]. However, for high normal strength and peel strength where directionality or easy off–on performance is not needed, this particular version of the biomimetic fibers tends to perform best.

Our unique contributions to these general types of mushroom-shaped adhesives included the introduction of multiheight caps [25] or asymmetric caps [26], which produce different directional adhesion without significantly altering the manufacturing process of the caps themselves (Figure 1), and multimaterial adhesives for different adhesion mechanisms in a single sheet. A deliberate defect to yield anisotropic adhesion was described in 2013 by my student Walid bin Khaled [25]. It has been thoroughly characterized in various papers, including his work and that of visiting student Yue Wang, who collaborated with me between 2015 and 2016 [27]. Yue Wang also developed the adhesion circle test system, which has been instrumental in characterizing how these fibers with the deliberate defect function, demonstrating that the same geometry with different structural materials can exhibit vastly different adhesion properties [28]. Generally, structural materials for biomimetic adhesives such as silicone rubbers, which behave more linear-elastically, show a dramatic difference in adhesion force with defects, whereas materials that are more viscoelastic, such as thermoplastic elastomers or polyurethanes, are less sensitive to small defects intentionally introduced into the cap structure [28]. The choice of material depends on the application requirements; silicone rubber may be preferable for applications needing high directionality and easy activation/deactivation, while materials like polyurethane or thermoplastic elastomers are better suited for tolerating slight surface roughness and are far more cost-effective.

**Figure 2:**
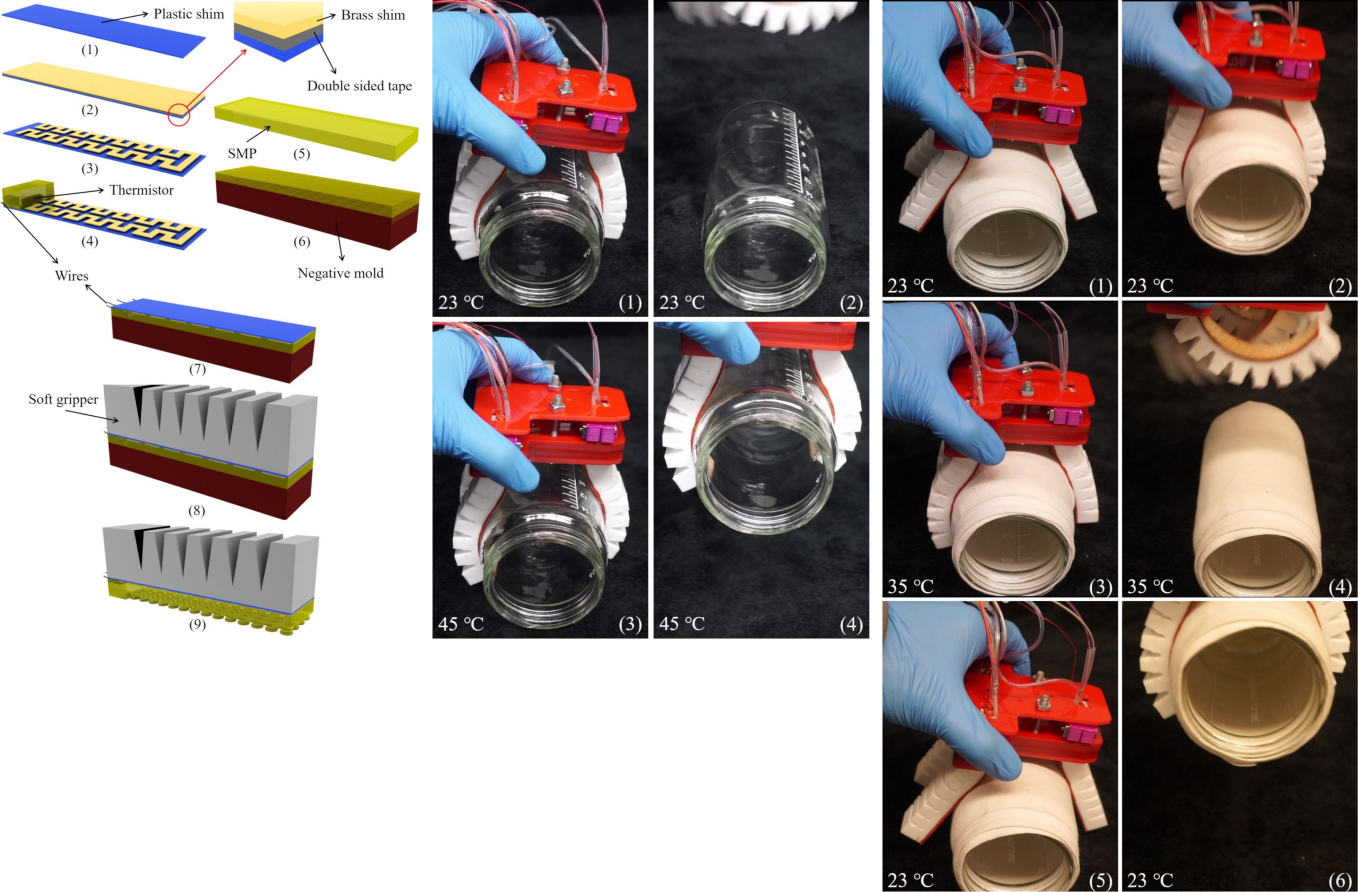
Compilation showing the assembly and integration of shape memory polymer dry adhesives with embedded heaters and soft robotic actuators. When in the soft state (*T* > 35 °C), the adhesive can grasp smooth surfaces but cannot support fabrics; when in the rigid state, it can pick up fabrics but not adhere to smooth glass. Compilation figure from [29] (T. Zhang et al., “Integration of Thermoresponsive Velcro-like Adhesive for Soft Robotic Grasping of Fabrics or Smooth Surfaces”; Proceedings of 2019 2nd IEEE International Conference on Soft Robotics (RoboSoft), published by IEEE. All rights reserved. Copyright © 2019 by IEEE. Reprinted with permission from IEEE. This content is not subject to CC BY 4.0.

The significant influence of mechanical properties on identical fiber designs has also been extensively studied by our group and others, including work on shape memory polymers (SMPs) for biomimetic pillars [29]. These uniformly mushroom-shaped SMP fibers could function as either a Velcro-like material or a biomimetic adhesive, depending on the modulus of the shape memory polymer [29]. Using MM3520, a shape memory polymer with a transition temperature of approximately 35 °C, and toggling between cool and warm states, the mechanical modulus could change by over two orders of magnitude. This allowed the material to function either as a soft rubber or as a rigid thermoplastic. Integrated with microheaters within a soft robotic gripper, the system demonstrated that fabrics could be grasped in its rigid state, while smooth surfaces like glass could be easily adhered to in its soft state (Figure 2). Thus, it served as a dual-mechanism dry adhesive, although it could not operate with both mechanisms simultaneously. SMP materials are finding increasing use in newer versions of dry adhesives [30–32], and their full capabilities for switchable adhesive materials are still to be determined.

**Figure 3:**
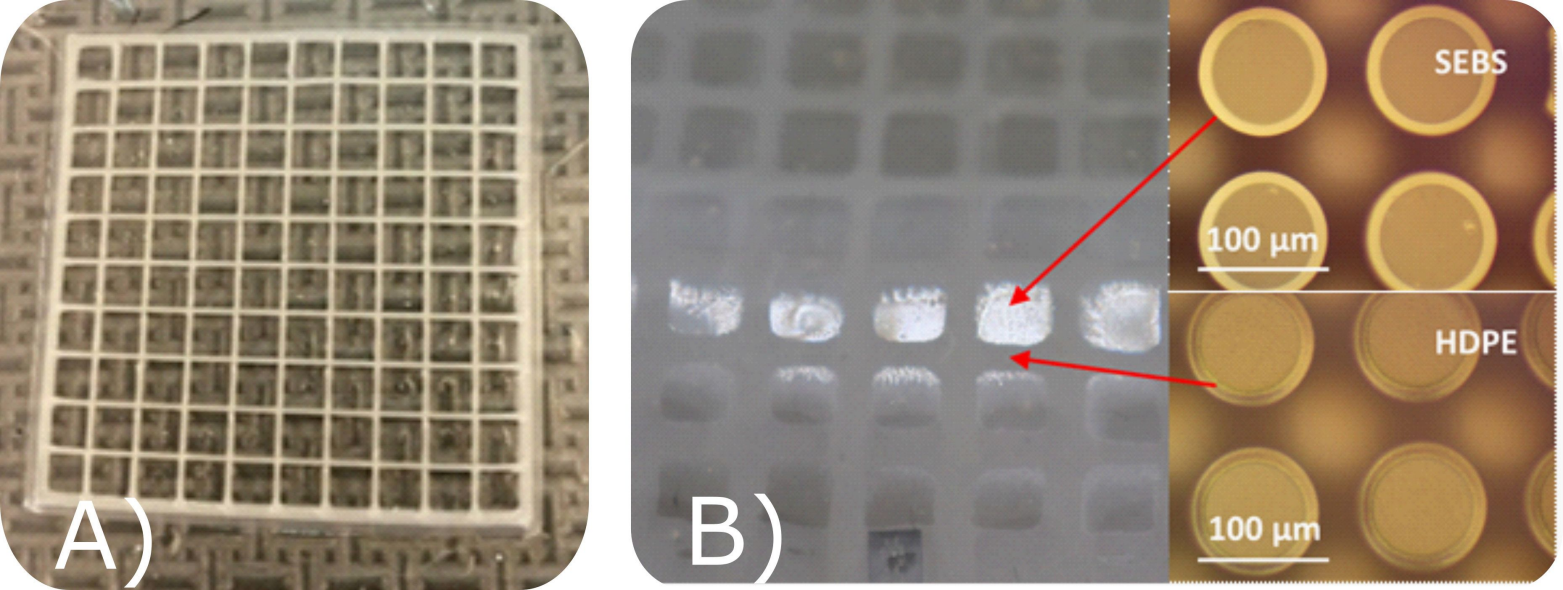
(A) 3D-printed grid of high-density polyethylene (HDPE) on top of a thermoplastic elastomer base. When melted together and formed into a gecko adhesive structure, a spatially heterogeneous material with fibers of the same shape (B) can simultaneously adhere to fabrics and smooth surfaces (see video in Supporting Information File 1).

Subsequent developments during the COVID-19 pandemic showed that multiple types of thermoplastic elastomers and thermoplastics could be combined into a single dry adhesive sheet with mechanically dissimilar materials forming the same fiber mold [33] (Figure 3). This innovation enabled high shear strength on fabrics and high normal strength on smooth surfaces simultaneously without needing to alter the adhesive temperature. Such material inhomogeneity represents an underexplored aspect of biomimetic dry adhesives and warrants further investigation. It also draws inspiration from nature, as it is quite common for many animals to include multiple adhesion mechanisms. Between multimaterial 3D printing and the use of newer techniques to precisely define micro/nanofeatures [34] that are beyond the capabilities of traditional lithography, there is a good deal left to accomplish for grippers, fasteners, and other adhesive mechanisms using biologically inspired techniques from more than one animal simultaneously.

**Figure 4:**
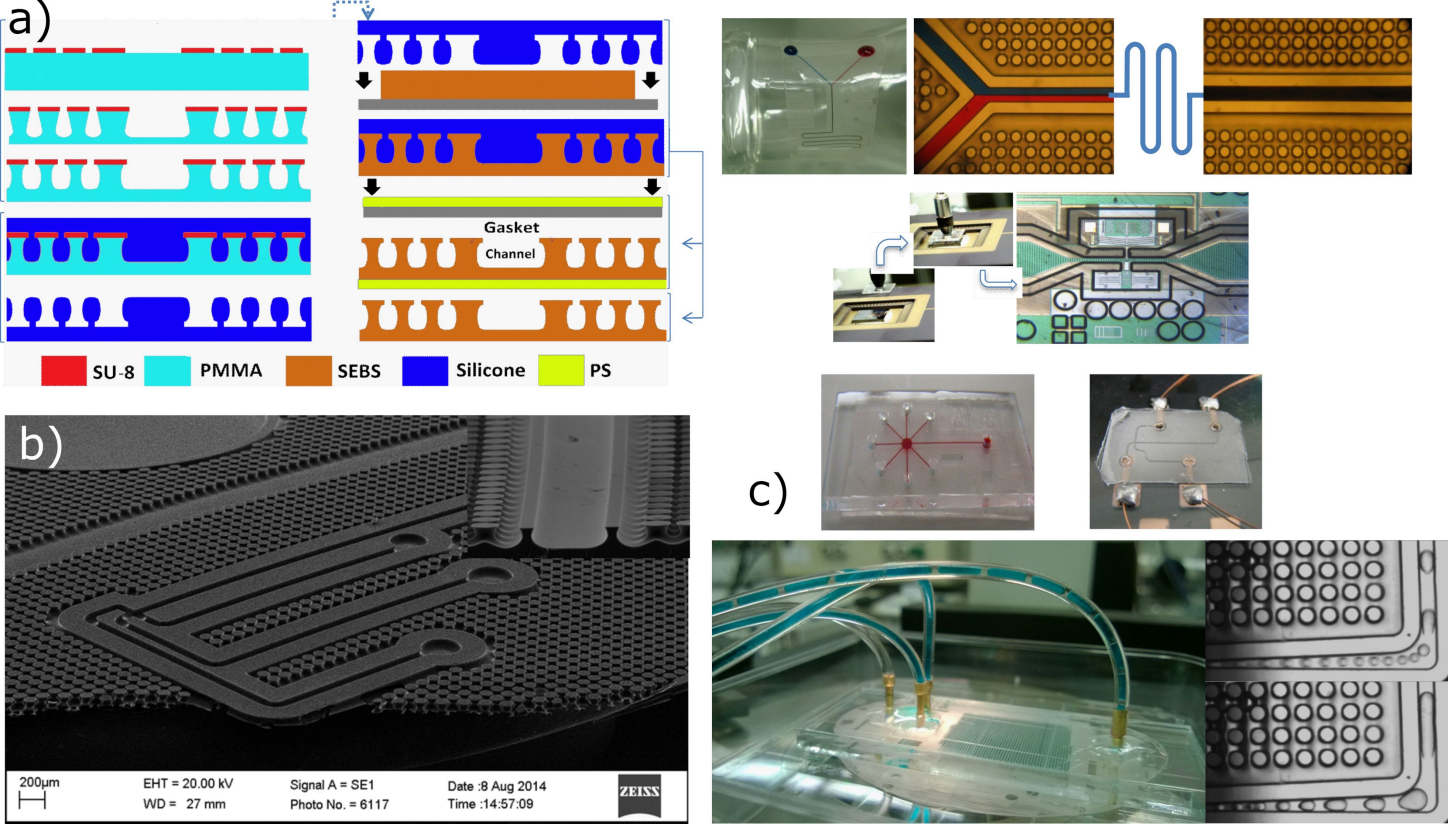
Geckofluidics process using similar crack-tolerant microstructures as gecko adhesives, but to define continuous gaskets for containing fluids. Figure 4 is a compilation of two figures from [35] (“Gecko gaskets for self-sealing and high-strength reversible bonding of microfluidics“) by A. Wasay and D. Sameoto, distributed under the terms of the Creative Commons Attribution 3.0 Unported License, https://creativecommons.org/licenses/by/3.0. The source journal is © The Royal Society of Chemistry 2015. Panel (a) represents the fabrication process that may include gasket structures in addition to fibers and rigid backing layers to ensure that pressure within channels is distributed between more fibers surrounding the channels. Panel (b) shows a scanning electron microscopy image of a 5 × 7 mm gecko gasket design for electrophoresis. Panel (c) is a compilation image of multiple applications for the gecko gaskets, including microfluidics integrated on non-planar surfaces, bonded to microelectromechanical systems (MEMS), and their use in droplet generation with oil and water.

Beyond merely serving as an adhesive surface, mushroom-shaped biomimetic fibers can be functional in ways entirely absent in nature. This concept was initially applied as an adhesion mechanism for microfluidics, a distinct project I was working on in 2012, that required a reversible adhesion system offering high strength, low contamination, no damage to mating surfaces, and no need for separate glues, plasma treatments, or magnets. A simple modification of the biomimetic fibers created a continuous gasket capable of containing fluids [35] (Figure 4). In addition to generating adhesion, these fibers were sufficient to confine fluids, such as oil and water, and gases within microfluidic channels at pressures up to approximately 90 psi. This approach demonstrated an order of magnitude increase in adhesion strength compared to the reversible bonding of PDMS and achieved this without any alterations to the material itself, merely the channel geometry was made to minimize crack propagation.

**Figure 5:**
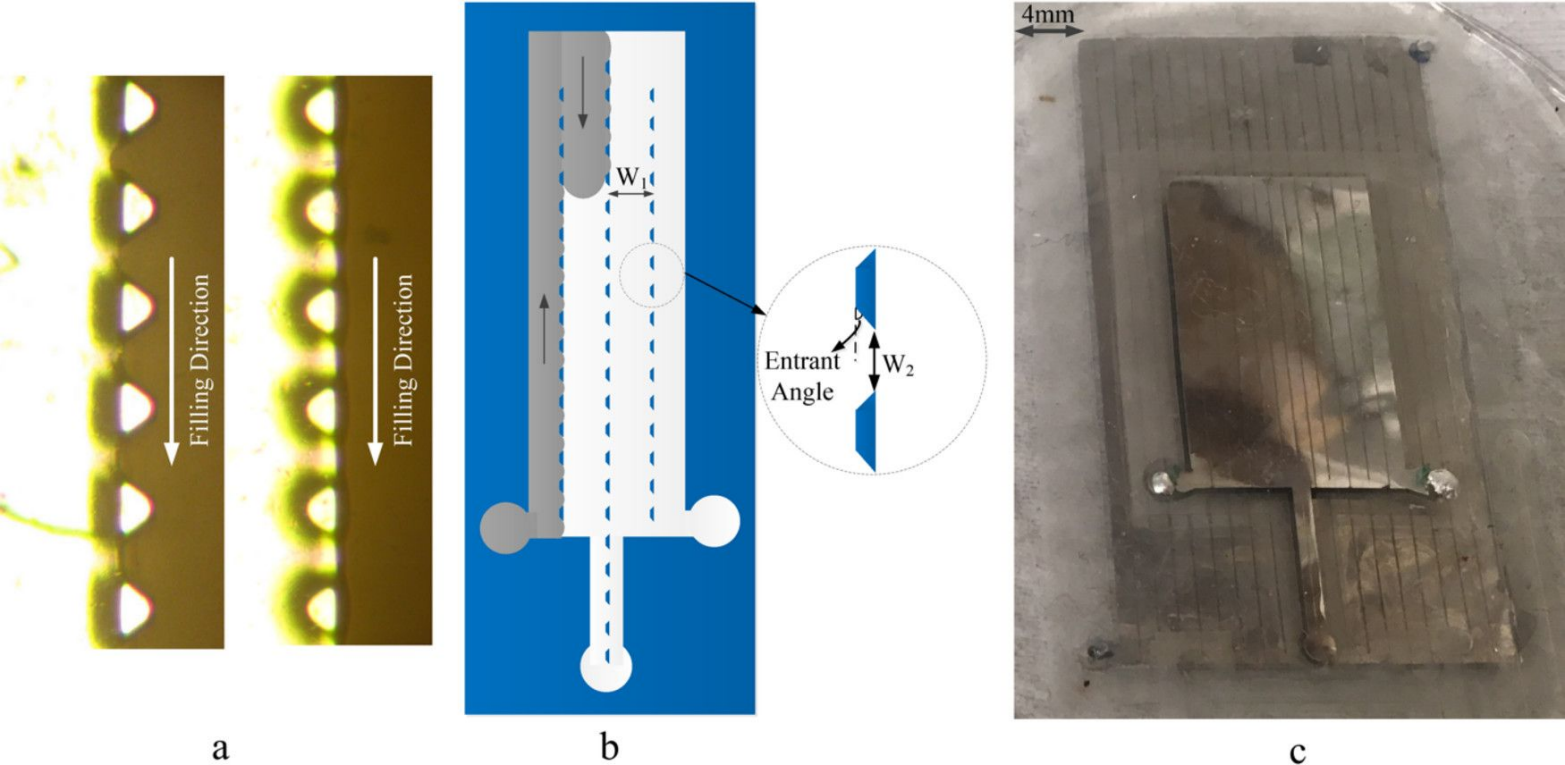
Laplace barriers within geckofluidic channels have directed room-temperature liquid metals (eutectic gallium–indium) with extremely high surface tension to properly fill complex microchannels (a, b) for the production of stretchable antennas (c) and electronics. Figure 5 was adapted from [36] M. Zandvakili et al., “Gecko-Gaskets for Multilayer, Complex, and Stretchable Liquid Metal Circuits and Antennas” Adv. Mater. Technol., with permission from John Wiley and Sons. © 2017 WILEY-VCH Verlag GmbH & Co. KGaA, Weinheim. This content is not subject to CC BY 4.0.

Although the original geckofluidics application targeted traditional microfluidics liquids like oil and water, another student in 2017 demonstrated its utility for integrating liquid metal electronics. Mersedeh Zandvakili showed in 2017 that eutectic gallium–indium could be injected into microfluidic channels, and the gecko pillars not only provided adhesion for the channels but also directed the liquid metal flow via Laplace barriers within the channels, offering a mechanism by which we could precisely control the filling of a liquid with very high surface tension [36] (Figure 5). Additionally, small subfeatures within the microfluidic channels could allow for both air escape and the addition of acid in specific locations of the biomimetic adhesives to direct the flow of liquid metal into separate, isolated areas. Handling such extreme materials is something no animal would have evolved to manage, yet the artificial versions have proven to be very functional in contact with these liquids and have enabled for a variety of flexible electronics applications.

While the geckofluidics project was an example of a traditional application enhanced through the use of an improved bonding solution, the key to advancing the utility of biomimetic adhesives lies in finding a slam-dunk application that drives investment into scalable manufacturing. While microfluidics has many uses, it is still a very small market even in comparison to traditional adhesives. Since 2017, however, we have been working in an area that is very different from what biomimetic adhesives have been applied to previously, and a very promising application is the development of shape- and stiffness-tunable composites.

Anyone familiar with the movies “The Terminator” or “Big Hero 6” has seen a sci-fi version of robots that can change their shape, size, or function. Science fiction offers numerous intriguing examples where versatility, customization, improved performance, and functionality result from shape- or stiffness-tunable materials. However, there are many real-world applications for shape- and stiffness-tunable materials beyond the realms of science fiction, for example, prosthetic sockets that interface with individuals with limb differences. Enhancing comfort requires the socket to precisely match the stiffness and shape of the body. However, there are significant differences between individuals and even within an individual over the course of their life, which should be easily accommodated with adaptable materials. Another, more mundane example could be the car body of a standard vehicle. Typically, a fender bender necessitates replacement of the bumper if the latter has been damaged. However, imagine if that bumper could simply “turn off and on again”, healing back to its original form. Nature offers many examples of self-healing damage over time, yet human-engineered products have rarely achieved this capability in a cost-effective manner [37]. Materials that can absorb energy and deform without being permanently damaged would be highly attractive from a sustainability perspective. A final example of practical products involves protective garments. Many elderly individuals wear hip protectors to guard against broken hips in the event of a fall [38]. However, these are often bulky, unattractive, and uncomfortable. If these materials could act as regular clothing that only transforms into body armor at the moment of a fall, their acceptance by the wider public would likely be much higher, reducing the health impact of traditional falls and injuries.

Academic literature is replete with instances of stiffness-tunable materials [15], and interest in this technology surged around 2010 with the advent of the modern soft robotics field. Traditional stiffness-switching mechanisms include phase change materials [39], jamming actuators [40], electrorheological fluids [41], and antagonistic actuators [42], but vacuum jamming has been the most popular in academic literature [43]. Vacuum jamming forces a series of particles, fibers, or sheets together to act as a semi-solid under vacuum. For instance, granular jamming can employ something as simple as coffee grounds in a rubber balloon to function as a shape-morphing actuator. Layer jamming, where different stacks of paper or thin film sheets combine under vacuum, offers more resilience and strength. However, vacuum jamming can present problems due to leaks, power consumption, noise from pumps, and the ultimate strength of the parts being limited by coefficients of friction and applied vacuum pressure.

**Figure 6:**
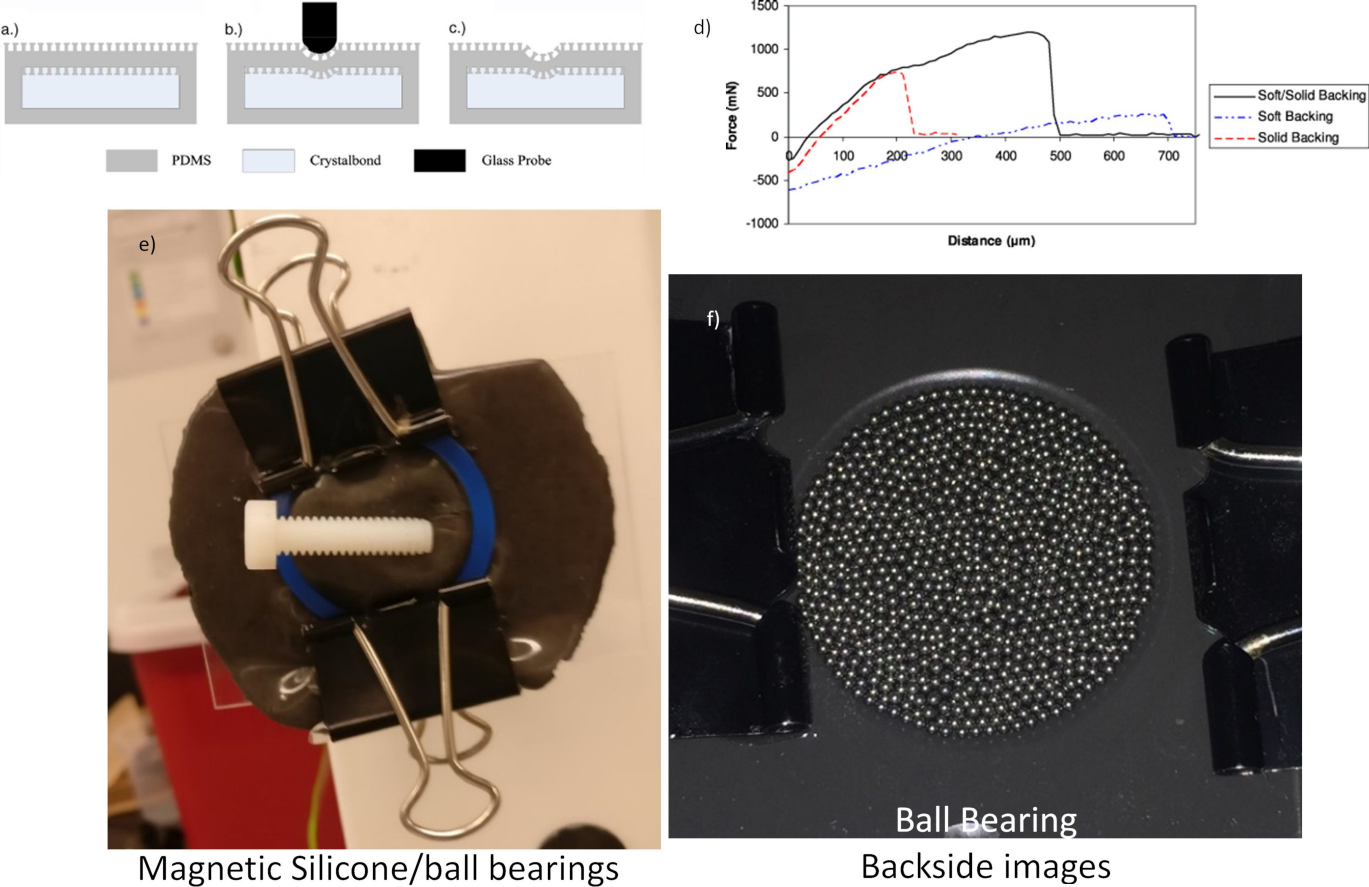
Non-vacuum jamming based stiffness-tunable prototypes developed for use in space applications. Figure 6a–d were republished from [39] (“Controllable biomimetic adhesion using embedded phase change material”, Smart Materials and Structures, vol. 20, article no. 015014, published on 9 December 2010; https://doi.org/10.1088/0964-1726/20/1/015014); © 2010 IOP Publishing. Reproduced with permission via Copyright Clearance Center. All rights reserved. This content is not subject to CC BY 4.0. Panels (e, f) are unpublished work with image credit and permission from Samuel Lehmann 2016 – this content is not subject to CC BY 4.0.

My interest in stiffness tuning dates back to the inception of our biomimetic adhesive projects for the European Space Agency. I quickly realized that a very sticky and soft gecko-inspired foot lacked the structural rigidity to support a load, and those that were very stiff proved exceptionally challenging to achieve good contact with surfaces and achieve large adhesive strength [21]. A material that was soft when making contact and rigid when supporting a load would be ideal, and in fact this is still an area where modern efforts continue [31,44–45]. Our closest demonstration at the time to achieve this stiffness tuning involved using an internal wax support structure within the biomimetic adhesives, acting as a soft interior in its semi-molten state and being very rigid when cooled [39]. However, the downside of thermally induced stiffness was the time scale required for modulus change, taking about five to ten minutes with materials we were working with back in 2010 (Figure 6a–c).

Alternatively, we considered magnetic jamming including ball bearings and magnetorheological fluids within silicone pouches mixed with magnetite (Figure 6e,f). Unfortunately, the weak link in these jamming mechanisms was bonding the pouch to the ball bearings or internal fluid as the magnetic silicone had relatively weak attraction and was located at the furthest location from the magnetic trigger (NdFeB magnets). Without a strong bond between the silicone membrane and the internal magnetic materials, the actuator could not support substantial adhesive loads, leaving these projects unpublished and remaining an internal curiosity.

During a sabbatical in 2017, I first encountered projects related to layer jamming in the George Whitesides Lab for soft robotics applications. I quickly realized that the layer jamming concept could be perfectly suited for biomimetic adhesives, as their coefficient of friction could be significantly higher than one, and our work on geckofluidics with rigid backing materials was already indicating similar capabilities. Once in contact with a smooth surface, isotropic biomimetic adhesives do not require power to maintain adhesion and can function in various relatively extreme environments while providing adhesion pressures up to several megapascals [34], which is an order of magnitude better than vacuum-based jamming under standard atmospheric conditions.

This revelation that biomimetic adhesives could in fact be best suited as the internal surface of a composite material sparked a new and exciting research direction for soft robotics and adaptable composites. The reasons to use these materials as an internal rather than an external surface can be broadly categorized as follows:

- Uniform contact surfaces: Unlike a gecko, the adhesive materials do not need to be overdesigned for potentially touching dirty, rough, wet surfaces but can always be in contact with an ideally suited surface.
- No contaminants: When contained within a composite, these adhesives can be almost entirely free of contaminants and remain so if there is an outer sleeve to keep dust and debris out.
- High bond strength: Theoretically, a biomimetic adhesive can exhibit far higher bond strength than vacuum jamming. Literature reports adhesion strengths greater than 1 MPa with optimization [34], whereas vacuum jamming can at best support approximately 100 kPa of normal load between individual sheets when operating under normal atmospheric conditions.
- No active vacuum or power needed: Once adhered, no continuous vacuum or power is required to maintain the jamming state, an attractive property for applications that maintain their state for long durations and only need to be reformed occasionally.
- Energy damping: Depending on the structural layers for the biomimetic adhesives, there could be significant internal energy damping for energy absorption, that is, highly viscoelastic fibers and energy dissipation as heat during deformation and adhesion failures may possibly provide better crash protection or armor functionality in wearable composites.
- Triggering mechanisms: The actuation of these materials can be combined with electrostatic forces or magnets to enhance overall functionality. Pneumatic pressures acting globally (for vacuum triggering) or locally (using positive pressure in geckofluidic channels) are also feasible.

**Figure 7:**
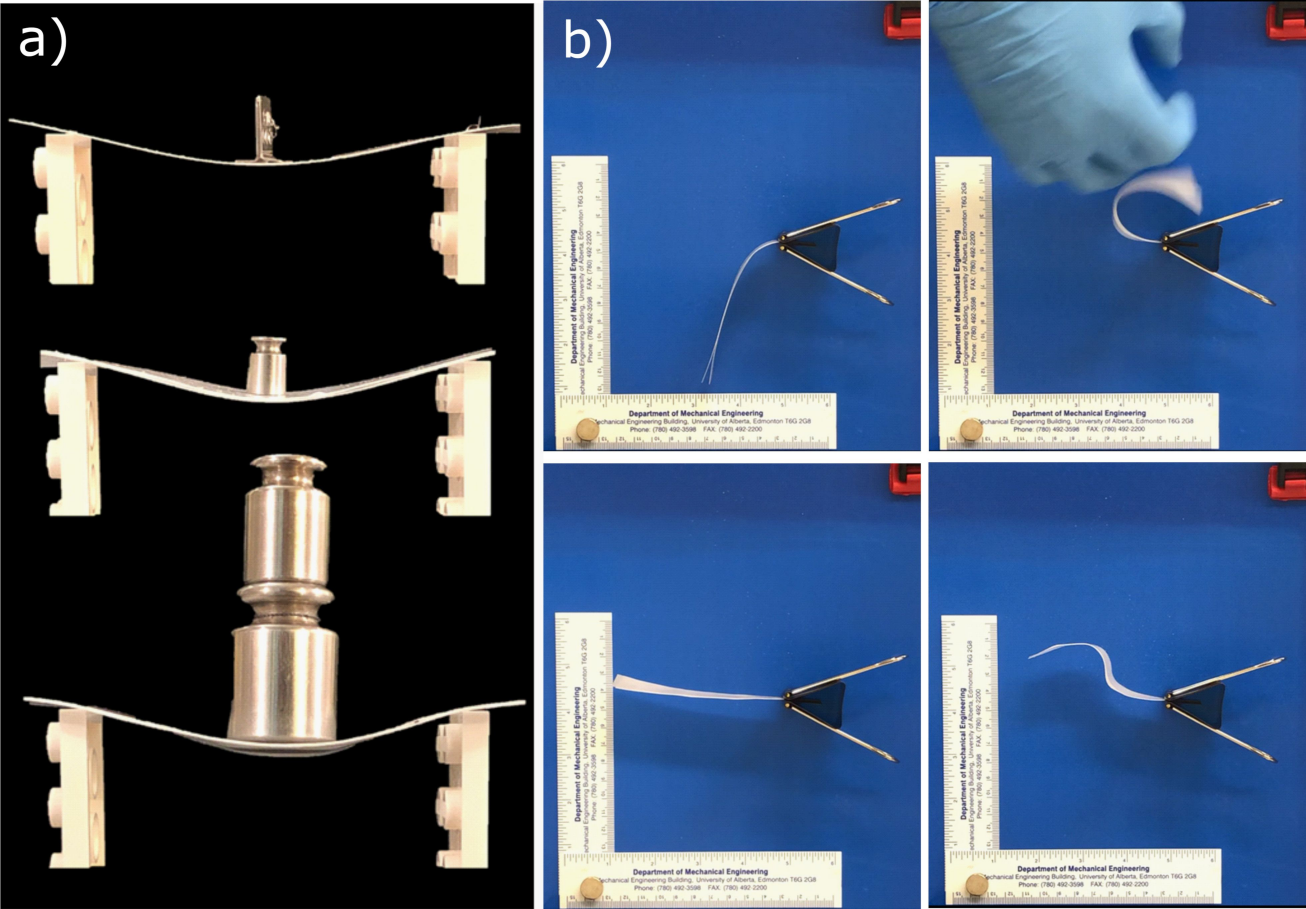
(a) Composite images of one and two-layer SETEX tapes prior to adhesion (top two images) and, at the bottom, a demonstration of how the bending stiffness increases with the adhered tapes. (b) Images taken as screen captures from a video demonstrating shape morphing/fixing capability of a gecko tape composite. Images are reproduced from [46] © The Adhesion Society. This content is not subject to CC BY 4.0.

The utility of multiheight fibers, understood back in 2008 based on personal observations of spider foot hairs’ adhesion mechanisms, is that a biomimetic adhesive can remain in a default non-adhesive state under light loads but become adhesive upon applying a certain minimum pressure level. This concept inspired the first generation of gecko-jammed composites presented at the Adhesion Society annual meeting in 2020 [46]. To achieve a cost-effective system, we opted for commercial biomimetic materials from Setex, modified to incorporate anti-adhesive features that we could typically produce in our custom processes. We applied cell disruption media, consisting of 100 µm diameter glass spheres, randomly or deterministically on sheets of these dry adhesives. The glass beads were large enough that the normal state of two sheets of dry adhesives would be non-adhesive unless a preload exceeding approximately 5 kPa was applied, at which point the fibers could self-adhere and prevent relative motion between the two sheets. The results were dramatic as these biomimetic composites did not merely act as two jammed sheets but rather as a sandwich composite. This significantly increased overall stiffness because the stiff material, that is, the backing layers, is supported a considerable distance from the core axis. Figure 7 shows demonstrations of how these materials can function statically for changing stiffness or dynamically so that the materials can be deformed and then fixed into multiple states. While no substantial work continued on this for several years because of the COVID-19 pandemic, we are now pursuing different optimized geometries, structural materials, and methods to control the internal adhesion for improved reconfigurability.

**Figure 8:**
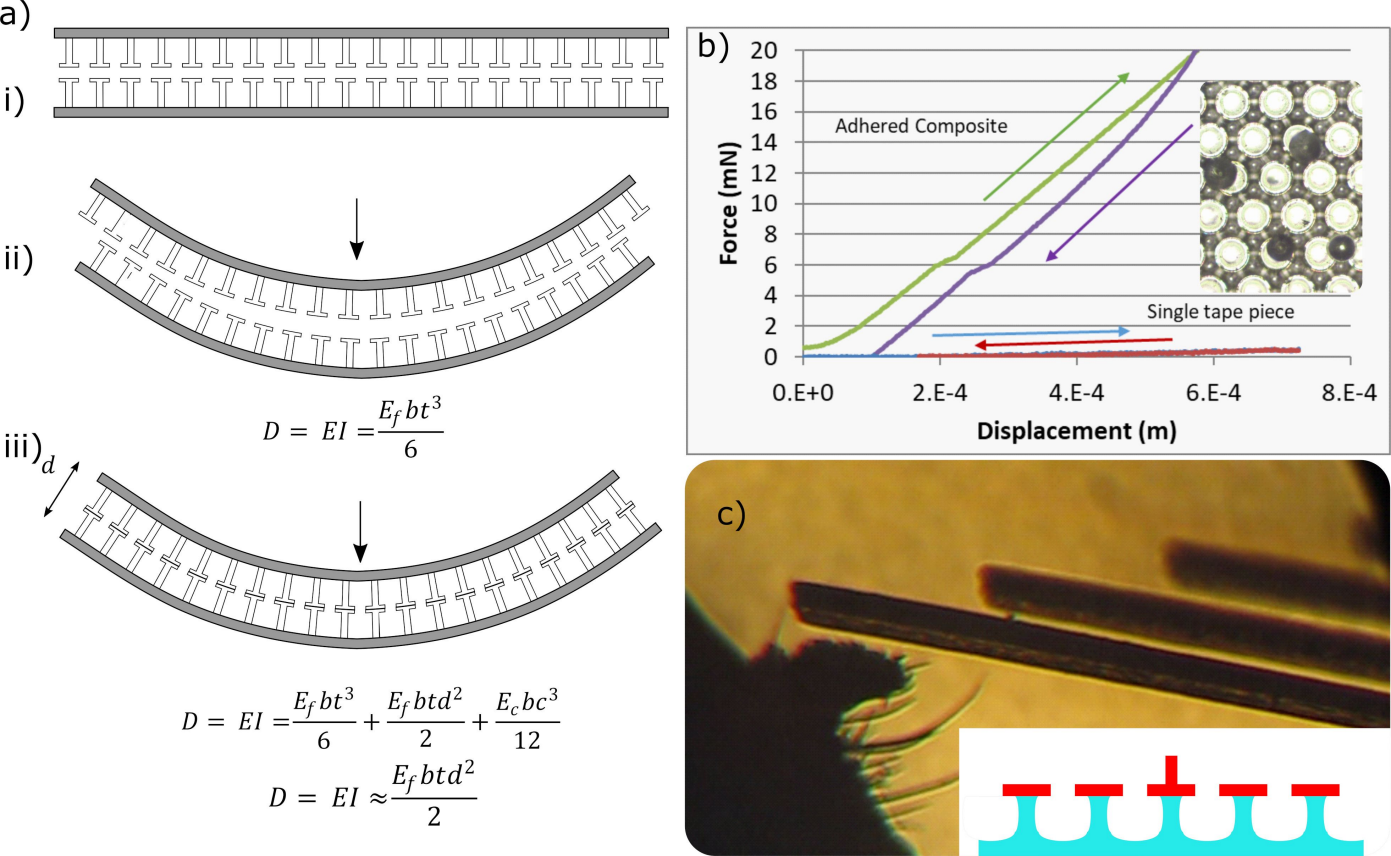
(a) i) Schematic of two gecko adhesives in their default non-adhesive state, ii) simplified modeling of effective bending stiffness as either two parallel non-adhesive sheets or as iii) a bonded sandwich composite structure. (b) Small scale tests show substantial bending stiffness increase as anticipated, but the large hysteresis indicates that internal delamination and plastic behavior can occur with these proof-of-principle designs. The inset shows the relative size of the anti-adhesive beads on top of the Setex surface. (c) Multiheight fibers inspired by spider legs (2008 experiments) as previously disclosed [48] may further enhance composite behaviors for on–off functionality.

Simple mathematical models and simulations can predict the overall effectiveness of these biomimetic adhesively jammed structures compared to traditional layer jamming with uniform layers of material (Figure 8). Information on the basic guidelines for sandwich composites can be found elsewhere [47]; the bending stiffness *D* is a function of the outer film modulus *E*_f_, the distance between the central axis of those films (defined by the core thickness *d* and the film thickness *t*), the modulus of the core *E*_c_, and the width of the composite *b.* If the “core” is made of independent fibers, it can have an effective *E*_c_ that is close to zero when in the unadhered state and will, therefore, minimally affect bending stiffness in comparison to two independent thin films of *E*_f_.

For the simplified model shown in Figure 8, if *E*_c_ ≪ *E*_f_ and *t* ≪ *d*, then the overall bending stiffness increase for the adhered composite compared to its unadhered state can be approximated as:


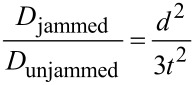


For a film thickness merely one tenth that of the core material, we would expect approximately a 30-times increase in bending stiffness, simply by bonding two pieces of biomimetic tape. In practice, this also works with materials like pressure-sensitive adhesive tapes. However, the assumption of negligible *E*_c_ is less appropriate, and their geometries are less suited for the purpose, resulting in lower bending stiffness increase per layer. Since the publication of this work, other similar mechanisms [49] have been demonstrated and may yet be improved by other groups working toward similar goals. Hence, the tuning of adhesion within composites could be an important area of investigation for years to come.

While this is a demonstration of bending stiffness increase, similar effects can be achieved regarding tension, much like an electrostatic clutch; in those instances, an even higher effective stiffness can be achieved in comparison to the unbonded state [50]. While work on this topic is just now restarting in our own group after disruption and delays from the COVID-19 pandemic, we hope to develop better designs suited for the purpose of on–off adhesion with bioinspired adhesives and apply them to morphing, stiffness-tunable composites for a variety of applications in the coming years.

## Conclusion

Gecko-inspired adhesives have had a long run in the academic literature, but the question of their future utility is at a crossroads: Do they remain a niche curiosity restricted to high-value, low-volume applications, or could they still become a breakout technology on par with Velcro® or the zipper? I personally hope that we can eventually push manufacturing and performance of these materials to such a low cost and high performance that it would be possible to build laminates, composites, clothing, and protective equipment out of these adhesive materials. We can ideally enable a type of van der Waals-based construction set for manufacturing products with strong, reversibly adhered laminates to improve adaptability, sustainability, and product performance. The future of reversible adhesives will come from looking within, and going beyond, biomimicry.

## Supporting Information

A demo of integrated HDPE fibers within an SEBS biomimetic adhesive sheet showing high shear strength (and negligible adhesion) on fabrics like spandex, while also being capable of having high peel strength on smooth plastic surfaces like polystyrene. The demonstration was completed in winter 2021 and presented at the Adhesion Society Virtual conference that year. Adhesive manufacturing, 3D printing, and demonstration was carried out by Dan Sameoto.

File 1 Multimaterial biomimetic adhesive demonstration.

## Data Availability

Data sharing is not applicable as no new data was generated or analyzed in this study.
